# FUNCTION-FOCUSED CARE FOR ACUTE CARE USING THE EVIDENCE INTEGRATION TRIANGLE: IMPACT OF PATIENT CARE ACTIVITIES

**DOI:** 10.1093/geroni/igad104.1119

**Published:** 2023-12-21

**Authors:** Elizabeth Galik, Barbara Resnick, Marie Boltz

**Affiliations:** University of Maryland School of Nursing, Baltimore, Maryland, United States; University of Maryland, Baltimore, Maryland, United States; Pennsylvania State University, State College, Pennsylvania, United States

## Abstract

The benefits of physical activity during hospitalization are particularly relevant for older adults living with dementia, as these individuals are at the greatest risk for functional decline, delirium, behavioral symptoms associated with dementia, increased length of stay, adverse events increased readmissions and institutionalization post discharge. One effective way to engage these patients in physical activity is through implementation of a function focused philosophy of care. Function focused care helps nurses to evaluate patient’s underlying physical capability and motivate them to participate in all care activities. The Function Focused Care for Acute Care Using the Evidence Integration Triangle study is a randomized controlled theoretically based trial that includes four components include: (1) Development of a Stakeholder Team; (2) Education of Staff; (3) Development of a Function Focused Care careplan; and (4) Mentoring and Motivating of Staff and Patients. Those randomized to control were exposed to education about Function Focused Care only. To date a total of 10 hospitals and 365 patients living with dementia have participated in the study. The mean age is 82, and the majority are female (62%) and white (72%). Although not statistically significant, there is evidence of improvement in the number of care interactions in which patients participated in the treatment versus control group. Cognition, quality of care interactions, behavioral and psychological symptoms associated with dementia, comorbidities, physical resilience, tethers and pain were all indirectly associated with performance of function focused care through performance of activities of daily living and/or pain.

